# Isolation, Characterization, Antioxidant Activity, Metal-Chelating Activity, and Protein-Precipitating Capacity of Condensed Tannins from Plum (*Prunus salicina*) Fruit

**DOI:** 10.3390/antiox11040714

**Published:** 2022-04-05

**Authors:** Liangliang Zhang, He Zhang, Lihua Tang, Xinyu Hu, Man Xu

**Affiliations:** 1 Institute of Chemical Industry of Forest Products, CAF, Nanjing 210042, China; zhangh@icifp.cn (H.Z.); tanglh@icifp.cn (L.T.); xinyuhu@icifp.cn (X.H.); xuman@icifp.cn (M.X.); 2 Key Laboratory of Biomass Energy and Material, Nanjing 210042, China; 3 Co-Innovation Center of Efficient Processing and Utilization of Forest Resources, Nanjing Forestry University, Nanjing 210042, China

**Keywords:** *Prunus salicina*, condensed tannins, antioxidant activity, MALDI-TOF MS, metal ions, protein-precipitating capacity

## Abstract

The type of polymeric condensed tannins from plum fruit (*Prunus salicina*) (PCT), the degree of polymerization and the distribution of polymers were characterized by MALDI-TOF MS and NMR spectroscopy. The metal-binding capacity of PCT with five metal ions (Cu^2+^, Zn^2+^, Al^3+^, Fe^2+^, and Fe^3+^) was characterized by a fluorescence quenching method. The results demonstrated the following: epicatechin was the basic unit occurring in PCT, and A-type and B-type linkages were the most common between the structural units of the polymers. The PCT have a strong antioxidant activity, which is comparable with that of the synthetic antioxidant BHA. The quenching mechanism of the PCT’s fluorescence intensity by Zn^2+^, Cu^2+^, and Al^3+^ was different from that of Fe^3+^ and Fe^2+^. Fe^3+^, Al^3+^ and Fe^2+^ had much higher affinities for the PCT than Zn^2+^ and Cu^2+^. A simple UV-Vis spectra method was developed to determine the protein-precipitating capacity of tannins. Bovine serum albumin (BSA) was effectively precipitated by tannins isolated from plum fruits, Chinese gallnut, sorghum grain, and *Platycarya strobilacea* at pH values between 4.5 and 5.0. A statistically significant linear relationship (*p* < 0.0001 or *p* < 0.0003) existed between the amount of tannin–protein complex formed and the amount of tannins added to the reaction mixture. The slopes of these lines indicated the protein-precipitating capacity of tannins.

## 1. Introduction

Tannins are a diverse group of plant-produced polyphenolic compounds, which are present in many plant foods, botanicals, and nutritional supplements. Many researchers have suggested that dietary tannin intake is associated with a decreased risk of coronary artery disease, cancer, urinary tract infections, and ulcers [1,2]. Tannins have received a lot of attention with respect to their possible nutritional and physiological actions. However, there are great variations in the structure and concentrations of tannins within and among plant species. Therefore, biomedical research on the evaluation of the health benefits and risks of increased tannin consumption is severely limited by the lack of suitable methods for the rapid characterization and standardization of these compounds.

**Figure 1:**
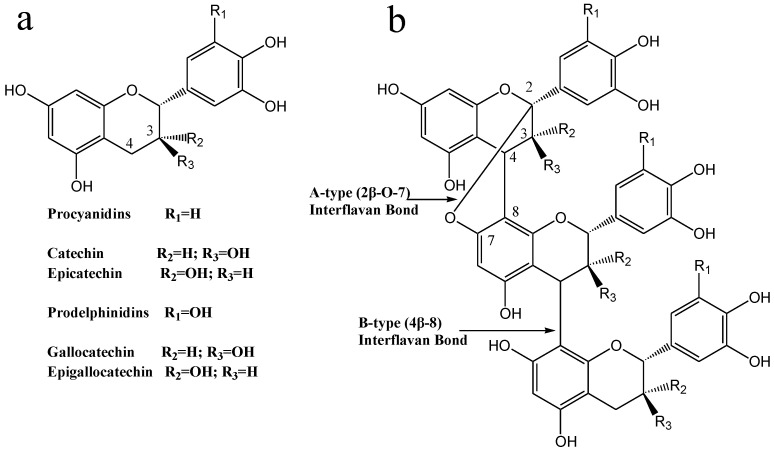
(**a**) Structures of the flavan-3-ol units. (**b**) Heteropolyflavan-3-ols from plum pericarp.

Tannins can be grouped into condensed and hydrolysable tannins, which are widely distributed in the plant kingdom in both food plants and in non-food plants. Hydrolysable tannins (HT) are classified as gallotannins or ellagitannins, and are polyesters of phenolic acids such as gallic acid or ellagic acid or their derivatives and D-glucose. The condensed tannins (CT) (also known as proanthocyanidins, PA) are polymers of flavan-3-ols, flavan-3,4-diols, or related flavanol residues linked via C4-C8 and/or C4-C6 linkages (B-type CT) or via C2-O-C7 and/or C2-O-C7 linkages (A-type CT) (Figure 1) [3]. CT can be divided into procyanidins, prodelphinidins, and propelargonidins, depending on the hydroxylation patterns of aromatic rings A and B of the flavan-3-ol units. The size of CT molecules can be described by the molecular weight (MW) as well as by the degree of polymerization (DP). Tannins are naturally occurring, water-soluble, and able to form complexes with proteins and polysaccharides [4]. CT have been reported to exert multiple biological effects as well as exhibit anti-inflammatory, antioxidant, anticancer, and antibacterial activities [5]. It has been suggested that the activities of CT depend on their antioxidant and chelating properties. CT are found to be effective metal ion chelators, and their excellent metal ion chelation properties are mainly associated with the galloyl and/or catechol group on the B-ring of flavan-3-ol units [6]. It has been demonstrated that metal ions interact with CT via ion exchange and complexation mechanisms.

Metal ions play an important role in many biological and environmental systems. Copper (Cu), zinc (Zn), and iron (Fe) are essential trace elements that have been studied in many diseases, including autoimmune, neurological, and psychiatric disorders [7]. Cu is involved in mitochondrial respiration, embryonic development, and neuronal functions [8]. Zn is involved in the formation of prostaglandins required for the maintenance of pregnancy and is present in the active centers of many enzymes. In plants, Fe is essential for mitochondrial electron transport and oxygen delivery [9]. However, when the concentrations of these biological metals exceed the proposed limit in bodies, there is high potential risk to humans and animals. For example, some transition metal ions can catalyze the Haber–Weiss reaction, which yields highly reactive and toxic radicals, which are associated with apoptosis through lipid peroxidation, as well as DNA and protein damage [10]. Therefore, it is essential to investigate the binding interactions of CT from common food sources and plants with these biologically important metal ions to provide dietary guidance for their nutritional intake by human beings and animals [11]. The possible mechanisms involved in the interactions of CT with these metal ions, including the main types of binding interactions, binding constants, binding sites, and main binding modes are still not fully understood. Aluminum (Al) is an environmentally important metal ion. Al is ubiquitously present in surface water and soil. Al is the most abundant metal in the Earth’s crust, comprising approximately 7% of its mass [12]. Higher levels of Al^3+^ solution are toxic to most plants, and thus studying the Al–tannin interactions could elucidate how plants regulate the bioavailability and toxicity of Al^3+^ through tannin exudation.

As a well-characterized model protein, bovine serum albumin (BSA) has been widely used to study protein–tannin interactions very recently [13,14]. By means of competitive binding and spectroscopic methods, previous tannin–protein interaction studies have confirmed that the protein structure is vital in forming the complexes. Generally, conformationally flexible proline-rich proteins show higher affinity to tannins because of the formation of particularly strong hydrogen bonds, and thus more accessible hydrogen-bonding sites [15]. The biological activities of CT depend on their chemical structure and concentration. However, due to their structural diversity and complexity, the qualitative and quantitative analysis of CT is a difficult task. Various techniques, including nuclear magnetic resonance (NMR), electrospray ionization-mass spectrometry (ESI-MS), and matrix-assisted laser desorption time-of-flight (MALDI-TOF) MS, have been used to characterize CT. Among these techniques, MALDI-TOF MS has proven to be highly suitable for the analysis of highly polydisperse and heterogeneous condensed tannins [16,17]. MALDI-TOF MS produces only a singly charged molecular ion for each parent molecule and allows the detection of a high mass with precision [16].

*Prunus salicina*, also known as Japanese plum, is an important fruit crop in China, and its pericarp has good taste and flavor. *Prunus* species have been used in traditional medicine for a variety of purposes [18]. The purpose of this study was to purify and characterize the CT of plum fruits (PCT) and to evaluate their antioxidant activity and metal-binding capacity. Furthermore, the protein-precipitating capacity of PCT was also investigated by UV-Vis spectra. The presence of condensed tannins up to DP13 as [M + Cs]^+^ containing up to two A-type linkages is first reported in this study. Proanthocyanidins in peanuts [19], cranberries [20], and other plant foods [21] occurring as A-type procyanidins and prodelphinidins were previously reported. Prunus tannins have unusual A-type linkages compared with the more common B-type linkages found in the CT from other tannin-rich foods [22].

## 2. Materials and Methods

### 2.1. Materials and Chemicals

The fruits of plum (*P. salicina*), at commercial maturation, were obtained from Yongtai, China. Fruits were stored at −20 °C prior to extraction. Frozen plum fruits (200 g) were extracted using acetone:water (7:3, by volume, 500 mL) at room temperature for 3 h. The extract was filtered through Whatman filter paper No. 1, and the filtrate was concentrated by a rotary evaporator at 40 °C. The concentrated extracts (100 mL) were loaded onto a Sephadex LH-20 chromatography column (30 cm × 2.5 cm), which was first eluted with methanol: water (50:50 *v/v*) including 2% acetic acid to remove oligomeric tannins and pigments, and then with acetone:water (7:3 *v/v*). The last fraction containing the PCT was freeze-dried overnight under a vacuum and stored frozen at −20 °C until further use. According to this method, tannins from Chinese gallnut (TA), *Sorghum bicolor* (Moench) grain (SCT), and the infructescence of *Platycarya strobilacea* (PSCT) were also extracted and purified.

A bovine serum albumin (BSA) solution (0.03 mM) was formulated with BSA (Sino-Biotechnology Company, Shanghai, China) in an acetate buffer solution (pH 3–6). Amberlite IRP-64 cation-exchange resin, l-ascorbic acid (AA), 1,1-diphenyl-2-picryl hydrazyl (DPPH), butylated hydroxyanisole (BHA), 2,2-azino-bis(3-ethylbenzothiazoline-6-sulphonic acid) (ABTS), and 2,4,6-tripyridyl-*_S_*-triazine (TPTZ) were purchased from Sigma-Aldrich Chemical Co. (St. Louis, MO, USA). Sephadex LH-20 was purchased from GE Healthcare Bio-Sciences (Uppsala, Sweden). Water was purified on Millipore Milli-Q apparatus. The working solution of the PCT (750 µg/mL) was prepared by dissolving tannins in a 50% methanol solution. The working solutions of Zn^2+^, Cu^2+^, Al^3+^, Fe^2+^, and Fe^3+^ were prepared by dissolving ZnCl_2_·6H_2_O, CuSO_4_·7H_2_O, AlCl_3_, FeSO_4_·7H_2_O, and FeCl_3_·6H_2_O, respectively, in double-distilled water or double-distilled water containing 0.1 M HCl to facilitate the dissolution of metal ions. The working solution of BSA (10 mg/mL) was prepared by dissolving BSA in water. All other reagents and solvents were of analytical reagent grade.

### 2.2. Estimation of Total Phenolics

The Prussian blue method was used to determine the total phenolics. The total phenolic content of plum pericarp was determined according to the method described by Price and Butler [23]. Briefly, 0.3 mL of the plum pericarp extract solution was diluted by 2.7 mL distilled water. Next, 1 mL of 8 mM K_3_Fe(CN)_6_ and 1 mL of 0.1 M FeCl_3_ in 1 M HCl were added into the solution and fully mixed. The mixture was placed at 24 °C for 15 min, then 3 mL of a 6.02 M phosphoric acid solution was added, followed by intensive mixing. After 2 min, 2 mL of a 1% Arabic glue solution was added and mixed well. The absorbance was read after 3–5 min in an Agilent Cary 8454 spectrophotometer (Agilent Technologies, Santa Clara, CA, USA) equipped with 1.0 cm quartz cells. A blank with an identical composition but omitting the plum pericarp extract, was analyzed and subtracted from all other readings. A standard curve was prepared using different concentrations (0–200 µg/mL) of tannins purified from plum fruit.

### 2.3. In Vitro Antioxidant Activity

The DPPH free-radical scavenging activity was measured using the method described by Ruan, Zhang, and Lin [24]. The scavenging activity of the ABTS radical was measured as described by Atere, Akinloye, Ugbaja, Ojo, and Dealtry [25]. The ferric reducing/antioxidant power (FRAP) assay was performed as described by Benzie and Strain [26].

### 2.4. NMR Analysis

For NMR characterization of the PCT, following our previously developed methods [27], samples were dissolved in a CD_3_COCD_3_/D_2_O (1:1) mixture and analyzed by ^13^C NMR (150 MHZ) spectroscopy using a Varian Inova-600 instrument (Varian, Palo Alto, CA, USA).

### 2.5. MALDI-TOF/MS Analysis

Our previously developed methods were followed [28]. The spectra were recorded in the positive ion mode on a Bruker Reflex III MALDI-TOF mass spectrometer (Bremen, Germany) using a matrix of 10 mg/mL 2,5-dihydroxy benzoic acid (DHB). A cesium trifluoroacetate (1.0 mg/mL) solution was mixed with the analyte/matrix solution at a 1:3 volumetric ratio to promote the formation of a single type of ion adduct ([M + Cs]^+^).

### 2.6. Fluorescence Quenching Assay

#### 2.6.1. Fluorescence Spectra

Our previously developed method [29] was used to record the fluorescence spectrum of the PCT in the range of 290–500 nm with an F96 fluorescence spectrometer (Lengguang Tech Inc., Shanghai, China). The excitation wavelength was set at 280 nm, and the emission wavelength was 314 nm. The excitation and emission slit widths were both set at 10 nm. All samples were measured in a quartz cuvette with a path length of 1 cm. Titrations were performed manually by using a micropipette. In each titration, the fluorescence spectrum was collected with the concentration of the PCT at 12.5 µg/mL. The experiments were repeated three times with similar results.

#### 2.6.2. Data Processing

Since the PCT showed UV absorption at 280 and 315 nm, the fluorescence intensity was corrected using the following (Equation (1)):

*F*_cor_ = *F*_obs_ × 10^(A1 + A2)/2^ (1)

where *F*_cor_ and *F*_obs_ are the corrected and observed fluorescence intensities, respectively, and A1 and A2 are the absorption of samples at the excitation and emission wavelengths, respectively [30].

The fluorescence quenching effects are described by the following Stern–Volmer equation (Equation (2)):

*F*_0_/*F* = 1 + *k*_q_*τ*_0_[Q] = 1 + *K*_sv_[Q] (2)

where *F*_0_ and *F* represent the fluorescence intensities of the fluorophore in the absence and presence of the quencher; *k*_q_ is the quenching rate constant in units of L mol^−1^ S^−1^; *K*_sv_ is the Stern–Volmer quenching constant; *τ*_0_ is the average lifetime of the fluorophore in the absence of the quencher, which is generally equal to 5 ns [31]; and [Q] is the concentration of the quencher.

The results were also analyzed by the modified Stern–Volmer equation (Equation (3)):

*F*_0_/Δ*F* = (1/(*f*_a_ × *K*_a_)) × (1/[Q]) + 1/*f*_a_ (3)

where Δ*F* = *F*_0_ − *F*; *F*_0_ and *F* are the integrated fluorescence intensities in the absence and presence of the quencher, respectively; [Q] is the concentration of the quencher; *K*_a_ is the modified Stern–Volmer quenching constant; and *f*_a_ is the fraction of fluorophore accessible to the quencher. The plot of *F*_0_/Δ*F* versus 1/[Q] yields a straight line with the slope 1/(*f*_a_ × *K*_a_) and the intercept 1/*f*_a_. The values of fa and *K*_a_ can then be calculated from the intercept and slope, respectively.

### 2.7. UV-Vis Spectra Study

The protein-precipitating capacity of tannins isolated from plum fruits, Chinese gallnut, sorghum grain, and *P. strobilacea* was determined by UV-Vis spectra. The effect of different purified tannin concentrations on the formation of CT–protein complexes was assayed. A series of methanolic solutions of purified tannin extracts (0.2–1.8 mg/mL) was prepared. The spectrophotometer was blanked with 900 μL of the reaction buffer solution (pH 3, 3.5, 4, 4.5, 5, 5.5, or 6) in a quartz cuvette with a path length of 1 cm. Next, 100 μL of the 10 mg/mL BSA and 100 μL of the different concentrations (0.4–2.0 mg/mL) of purified tannin solutions were added to the cuvette. The UV-Vis spectrum of the mixture was recorded after 1 min of mixing. The plot of the absorption values of mixtures measured at 510 nm versus the concentrations of purified tannin extracts yielded a straight line, and the slope could be interpreted as the protein-precipitating capacity. The effect of pH on the formation of tannin–protein complexes was monitored as described by NaczK, Oickle, Pink, and Shahidi [32].

## 3. Results and Discussion

### 3.1. Quantification of the Total Phenolic Content

The plum pericarp extract that was purified on the Sephadex LH-20 column gave a positive reaction in a butanol–HCl assay, indicating the presence of CT. Quantification showed the presence of a high amount of total phenolics (8.24% *w/w*) in the plum pericarp and in dry matter calculated as tannins purified from plum pericarp.

### 3.2. Antioxidant Activities of the PCT

**Figure 2:**
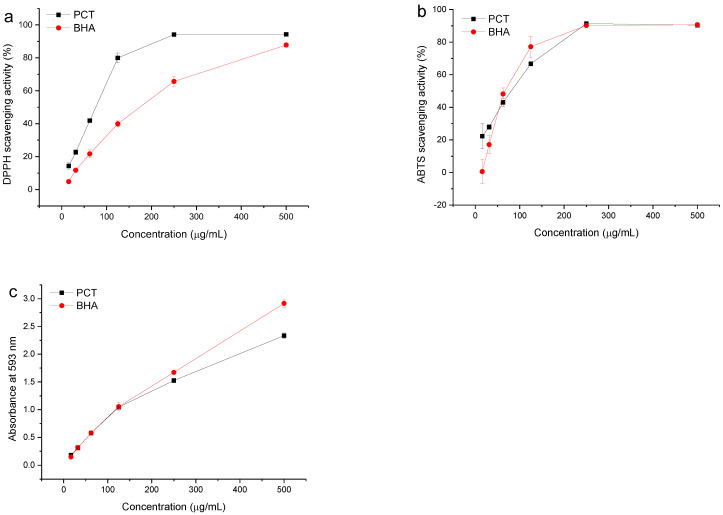
(**a**) DPPH and (**b**) ABTS free radical scavenging activities of different concentrations of tannins extracted from plum pericarp. (**c**) Ferric reducing power of different concentrations of tannins extracted from plum pericarp. PCT, condensed tannins of plum pericarp; BHA, butylated hydroxyanisole.

The free radical scavenging activities of PCT and the reference standard BHA were determined by the DPPH assay. A lower IC_50_ value indicates a higher antioxidant activity. The IC_50_ value of PCT (81.16 ± 3.16 µg/mL) was superior to that of the reference BHA (169.62 ± 11.36 µg/mL). Moreover, the activity was directly proportional to the concentration of tannins in the investigated sample (Figure 2a). An increase in the DPPH-scavenging activity in a concentration-dependent manner indicated that the PCT possesses potent free radical scavenging activity.

The antioxidant activity of the investigated extract was also evaluated by the ABTS method. The analyzed extract reduced stable blue-green ABTS^+^ to its colorless form. The IC_50_ of the PCT was 82.90 ± 3.57 μg/mL, which is very close to the value obtained by the DPPH assay and superior to that of the reference BHA (88.94 ± 4.75 µg/mL). Furthermore, the activity was directly proportional to the concentration of tannins (Figure 2b).

The FRAP values of the PCT at different concentrations are shown in Figure 2c. A higher absorbance corresponds to a higher ferric reducing power. The results indicated that condensed tannins from plum increased the ferric reducing power with increasing concentrations. The ferric-reducing antioxidant potential of the PCT was 5.83 ± 0.21 mmol AAE/g, which is very close to the value of BHA (5.89 ± 0.33 mmol AAE/g). Proanthocyanidin pentamers (200–170 mg/100 g) were found the main polyphenolic compounds in the ethanolic extract from Japanese plum skin and flesh [33]. Furthermore, this proanthocyanidin extract showed antioxidant capability (DPPH and FRAP assays) and cellular protection. All these data indicated that the proanthocyanidin obtained from plum can be useful as natural antioxidant additives and ingredients for functional food preservation.

### 3.3. NMR Analysis

The purified CT samples from plum fruit were characterized by ^13^C NMR spectroscopy, and the tentative assignment of the single resonances in the ^13^C NMR spectrum of CT was performed according to Zhang and Lin [27]. Two distinct signals at 145.0 and 145.4 ppm were observed in the ^13^C NMR spectrum (Figure S1), and they were assigned to the C3′ and C4′ in the catechin/epicatechin units of procyanidin (PC). The presence of the dominant procyanidin unit of the polymeric condensed tannins from plum fruit was further confirmed by the presence of strong peaks at 114.0–115.2 and 118.2–119.0 ppm, assigned as the C2′, C5′, and C6′ chemical shifts, respectively, of the catechol group in the B-ring. The presence of a clear signal for the ^13^C NMR chemical shift in condensed tannins from plum suggested that they are mainly composed of procyanidin units (catechin/epicatechin). Indeed, prodelphinidin units (gallocatechin/epigallocatechin) generally showed a typical resonance at 146 ppm [34]. No such chemical shift in the PCT ^13^C NMR spectrum indicated that they are predominantly composed of procyanidin units.

The spectral region between 70 and 90 ppm is sensitive to the stereochemistry of the C-ring of flavan-3-ol units. The 2,3-*cis*/2, 3-*trans* stereochemistry ratio could thus be determined through the distinct differences in their respective C2 chemical shifts [27]. The C3 of both *cis-* and *trans*- isomers displays a ^13^C NMR signal at 73 ppm, whereas C2 gives a resonance at 76 ppm for the presence of the *cis*-form and at 84 ppm for the *trans*-form. No such latter signal peak in the spectrum suggested that the PCT polymers only consisted of (−)-epicatechin as the units. The results thus showed that the PCT mainly consisted of procyanidins, which were composed of (−)-epicatechin as the main constitutive monomer. This is the first time the constitution of the polyphenols of plum pericarp has been elucidated.

The characteristic ketal carbon at 100 ppm in the ^13^C NMR spectrum suggested the presence of a doubly-linked (A-type and B-type) subunit in the structure of the PCT. The clear signals observed at 28.3 and 29.7 ppm were attributed to the C-4 carbons of the extension units and terminal units, respectively. Consequently, the signal observed at 36.4 ppm was assigned to the C-4 carbon of the middle units. The most downfield signals from 144.2 to 157.5 ppm were assigned to the phenolic carbons 5, 7, and 1′ of each unit as well as the C8a of each unit. The signals from 95.3 to 106.4 ppm could be assigned to carbons 6, 8, and 4a.

### 3.4. MALDI-TOF/MS Analyses

**Figure 3:**
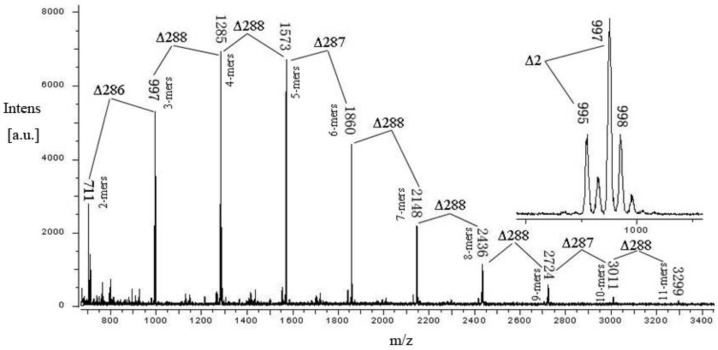
MALDI-TOF (positive reflectron mode) mass spectrum of condensed tannins from plum pericarp. Masses represent the epicatechin homopolymer of the polyflavan-3-ol series [M + Cs]^+^. The insert is the enlarged spectrum of the polyflavan-3-ol trimer. ∆ means value difference.

Figure 3 shows the MALDI-TOF mass spectrum of the PCT, which was detected as [M + Cs]^+^ adducts, showing that the PCT consisted of a series of repeating procyanidin (catechin/epicatechin) units. The results indicated that the PCT is characterized by a MALDI-TOF mass spectrum with a series of peaks with distances of 288 Da, corresponding to a mass difference of one catechin/epicatechin unit between each procyanidin polymer. Therefore, the polymeric condensed tannins were proposed to be composed of monomeric units with a molecular mass of 288 Da, and the prolongation of polymers was due to the addition of catechin/epicatechin monomers. Moreover, given the absolute masses corresponding to each peak, it was further suggested that the PCT contains only procyanidin polymers, as was already indicated in the respective ^13^C NMR spectrum. The mass spectrum showed a series of polyflavan-3-ols extending from the dimer (m/z 711) to the tridecamer (m/z 3875) in the positive reflectron mode (Table 1).

Based on the structures of the procyanidin polymers described by Zhang and Lin [27] an equation was formulated to predict higher polymerized heteropolyflavan-3-ols, and the data are shown in Table 1. The predictive equation is 290 + 288a + 133, where 290 is the molecular weight of the terminal epicatechin unit, a is the degree of polymerization contributed by the epicatechin extending unit, and 133 is the atomic weight of cesium. Application of this equation to the data obtained in the present experiment revealed the presence of a series of polymer CTs consisting of well-resolved procyanidin oligomers. In addition, a series of compounds that were ⊿2 amu multiples lower than those described in the predictive equation for heteropolyflavan-3-ols was also observed in the mass spectrum (Figure 3). This series of masses might represent polymer CT, in which the A-type interflavan ether linkage occurs (4–8, 2-O-7) between adjacent flavan-3-ol subunits, because two hydrogen atoms (⊿2 amu) are lost in the formation of this interflavan bond (Table 1). It has been reported that cranberries, a fruit that contains A-type linkages of polymer CT, also have a similar mass distribution [35]. For the first time, a compositional analysis of CT polymers from plum pericarp sources using MALDI-TOF/MS has been successfully demonstrated. Based on this technique, the average degree of polymerization was calculated to be 5.3, and the mean molecular weight was calculated to be 1583.7 Da. This is in agreement with the result of the previous study that proanthocynidin, with the most epicatechin extension units and a mean degrees of polymerization of 5.1 and 5.2, was found in Japanese plum peel and pulp, respectively [33]. Variations in the proanthocyanidins in epicarp and mesocarp tissue of Japanese plums during maturation and storage have been studied and indicated that differences were significant by cultivar and tissue type, and the degree of polymerization, as indicated by vanillin/proanthocyanidin ratios, did not increase in either cultivar with maturity or storage time. In this work, the structure of proanthocyanidin in Japanese plum was characterized by MALDI-TOF MS and NMR spectroscopy for the first time [36].

In addition, in order to obtain an ideal mass spectrum of tannin polymers, several factors, including the selection of an appropriate matrix, the optimal mixing, and optimal selection of the cationization reagent, must be optimized to develop MALDI-TOF/MS techniques. In the present study, Cs^+^ was selected as the cationization reagent, and it resulted in the best conditions for the MALDI-TOF analysis of CT polymers and gave a relatively simple MALDI-TOF spectrum.

### 3.5. Quenching of the PCT Fluorescence Spectrum by Metal Ions

**Figure 4:**
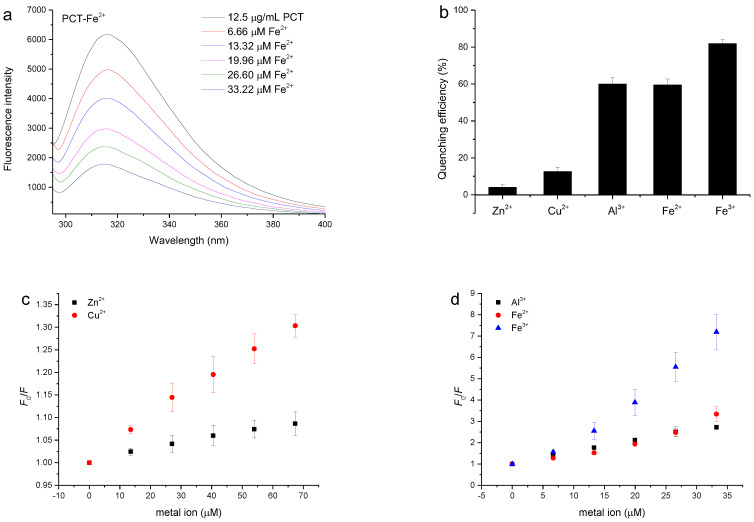
(**a**) Quenching of fluorescence intensity in 12.5 µg/mL polymeric condensed tannins from plum fruit (PCT) by Fe^2+^. Changes in the emission of the PCT were measured in a pH of 6.0, achieved with an acetate buffer; *λ*_ex_ = 280 nm. (**b**) The quenching efficiencies of 26.60 µM Zn^2+^, Cu^2+^, Al^3+^, Fe^2+^, and Fe^3+^ on the PCT were compared. The quenching efficiency was obtained by plotting [(*F*_0_ − *F*) × 100/*F*_0_] versus the metal ion concentration. (**c**,**d**) Stern–Volmer plots for PCT fluorescence quenching by Zn^2+^, Cu^2+^, Al^3+^, Fe^2+^, and Fe^3+^.

The interactions between metal ions and the PCT were studied using fluorescence spectroscopy [37]. Fluorescence quenching was the decrease in the quantum yield of the fluorophore caused by a variety of molecular interactions with quencher molecules, such as ground state complex formation, molecular rearrangement, energy transfer, and excited-state reactions [38]. The fluorescence of condensed tannins originated from its structural units, including (–)-epigallocatechin (EGC) and (–)-epicatechin (EC) units [6]. As previously reported by Zhang, Xu, Wang, and Hu [37], *Sorghum bicolor* Moench proanthocyanidin showed a strong fluorescence emission with a peak at 320 nm based on the excitation wavelength, and its fluorescence intensity gradually decreased with the addition of Zn^2+^, Cu^2+^, and Al^3+^. This was also observed in our experiment when studying metal ions titrated into the PCT solution. Figure 4a shows the attenuation of PCT fluorescence when Fe^2+^ was continuously added to 12.5 µg/mL PCT solution at a pH of 6.0 achieved with an acetate buffer, and this attenuation was also observed when Zn^2+^, Cu^2+^, Al^3+^, and Fe^3+^ were titrated into the PCT solution (Figure S2).

Based on the PCT fluorescence quenching spectra of the metal ions, the quenching efficacies of 26.60 μM metal ions on tannins are calculated and shown in Figure 4b. It can be seen from the graph that 81.78%, 59.94%, and 59.50% of the fluorescence intensity of the PCT was quenched by Fe^3+^, Al^3+^ and Fe^2+^, respectively, at a concentration of 26.60 μM. Meanwhile, only 3.98% and 12.58% of the fluorescence intensity of the PCT was quenched by Zn^2+^ and Cu^2+^, respectively, at the same concentration. These results indicated that Fe^3+^, Al^3+^, and Fe^2+^ are more effective quenchers for PCT than Zn^2+^ and Cu^2+^.

Representative Stern–Volmer plots for the fluorescence quenching of the PCT by the studied metal ions are shown in Figure 4c,d. As shown in the curves, the Stern–Volmer plots show an obvious deviation toward the *x*-axis when high concentrations of Zn^2+^, Cu^2+^, and Al^3+^ are added to the tannins. For these metals, the *K*_a_ was evaluated using the modified Stern–Vollmer equation (Equation (3), Figure S3a) and were shown in Table 2 to be appropriate for systems with two fluorophores with different accessibilities [38].

In contrast, the Stern–Volmer plots largely deviated from linearity toward the *y*-axis at high concentrations of Fe^2+^ or in Fe^3+^ mixed with the PCT (Figure 4d), indicating that not only dynamic quenching but also static quenching by Fe^2+^ and Fe^3+^ was involved in the interaction with tannins. The nonlinear Stern–Volmer plots that show a slight upward curvature indicate a combination of dynamic and static quenching processes. For the system in which both dynamic quenching and static quenching mechanisms are involved in the quenching processes, the Stern–Volmer equation can be written as in Equation (4), which, upon rearrangement, takes the form of Equation (5) [36].

*F*_0_/*F* = (1 + *K*_D_[Q])(1 + *K*_S_[Q]) (4)

[(*F*_0_/*F*) **−** 1](1/[Q]) = (*K*_D_ + *K*_S_) + *K*_D_*K*_S_[Q (5)

where *F*_0_ and *F* represent the fluorescence intensities of the fluorophore in the absence and presence of the quencher; *K*_S_ and *K*_D_ are the static and dynamic quenching constants, respectively, and [Q] is the concentration of quencher. The *K*_S_ and *K*_D_ values can be obtained by plotting [(*F*_0_/*F*) − 1](1/[Q]) versus [Q] (Figure S3b). The *K*_a_ of Fe^2+^ or Fe^3+^ quenching of the PCT’s fluorescence can be obtained by the intercept of the equation (*K*_S_ + *K*_D_), and the values are shown in Table 2. The results indicated that the affinities of Al^3+^ and Fe^3+^ for the PCT were much higher than those of other metal ions at a pH of 6.0. The affinities of the metals for the PCT could be ranked in the order Al^3+^ > Fe^3+^ > Zn^2+^ > Fe^2+^ > Cu^2+^, suggesting that trivalent metal ions exhibit higher affinities for condensed tannins than divalent metal ions.

To elucidate the effect of the pH on the binding reaction between metal ions and condensed tannins, the quenching of the fluorescence of condensed tannins from plum by Al^3+^ or Fe^3+^ at different pH values was also studied. Figure S4 shows a similar attenuation of the PCT fluorescence when Al^3+^ or Fe^3+^ was continuously added to 12.5 µg/mL PCT solution at a pH of 2.1 or 7.4. Changes in pH did not affect the fluorescence spectrum of the PCT. At all of the pH values tested, the PCT fluorescence was affected by Al^3+^ or Fe^3+^ in a dose-dependent manner. As shown in Figure S5 of the Supplementary Materials, the efficacy of metal-induced quenching depended largely on the pH values. At a pH of 2.1, 26.60 μM Al^3+^ quenched 17.4% of the PCT fluorescence. At a pH of 6.0, the same concentration of Al^3+^ quenched 60.0% of the PCT fluorescence. When the pH was increased to 7.4, 35.7% of the PCT fluorescence was quenched by 26.60 μM Al^3+^. Similar changes were also observed for PCT fluorescence quenching by Fe^3+^.

The Stern–Volmer plots of Fe^3+^-induced quenching of the PCT fluorescence at pH values of 2.1 and 7.4 were linear. Similar results were also found in the Al^3+^-induced quenching data at a pH of 7.4, whereas at a pH of 2.1, the data obtained showed nonlinearity, which was consistent with two fluorophores with different accessibilities (Figure S3c). The data of Al^3+^-induced quenching fluorescence at a pH of 2.1 yielded a linear modified Stern–Volmer plot, and the Stern–Volmer constant (*K*_a_) was calculated (Figure S3d). As can be seen from the graph, pH values greatly affect the binding interactions between metal ions and condensed tannins of plum. Moreover, the binding forces between the PCT and Al^3+^ and Fe^3+^ were the strongest at a pH of 6.0.

### 3.6. Protein-Precipitating Capacity Assay

**Figure 5:**
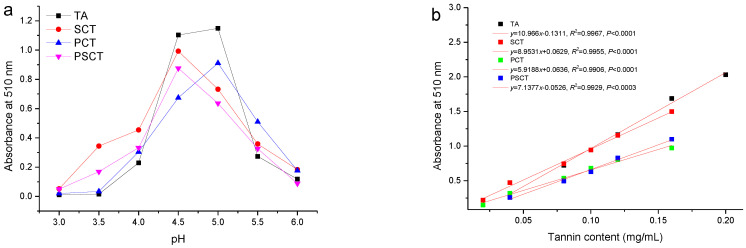
(**a**) The pH dependence of tannin complex formation with BSA determined by UV-Vis spectra. (**b**) Titration curves of a known amount of protein with increasing amounts of tannins of Chinese gallnut, plum fruits, sorghum grain, and *P. strobilacea*. TA, tannins isolated from Chinese gallnut; PCT, tannins isolated from plum fruits; SCT, tannins isolated from sorghum grain; PSCT, tannins isolated from *P. strobilacea*.

For determining the ideal pH for BSA tannin precipitation, tannins extracted from plum fruits, Chinese gallnut, sorghum grain, and *P. strobilacea* were treated at different pH, and the impact on their protein-precipitating capacity was investigated. Note that the ideal pH is the value at which a tannin–protein complex forms the greatest amount of precipitation. Figure 5a illustrates the effect of pH on the precipitate when BSA reacts with the purified tannins via the protein precipitation assay. It indicates that BSA was effectively precipitated at pH 4.5–5.0 after the tannins were added. Naczk et al. [39] reported a similar pH effect for condensed tannins obtained from canola hulls, evening primrose, and faba bean.

As shown in Figure 5b, with an increased amount of purified tannins added to a solution of 1 mg/mL protein, a tannin–protein complex was formed. There was a statistically significant (*p* < 0.0001 and *p* < 0.0003) linear relationship between the tannin–protein complex and the tannins in quantitative terms for up to 0.2 mg/mL and 0.16 mg/mL for tannins from plum fruits, Chinese gallnut, and sorghum grain. A similar relationship (*p* < 0.0003) exited for up to 0.16 mg/mL tannins from *P. strobilacea*. The slopes of the titration curves varied from 5.9 to 11.0 due to the different protein-precipitating capacities of tannins from different plants. These results suggested that compared with tannins from sorghum grain, *P. strobilacea*, and plum fruits, tannins from Chinese gallnut performed better in terms of protein precipitation. With this method, the protein-precipitating capacities of different plant tannin extracts can be determined. Importantly, the chemical structures, molecular weights, and polymer chain lengths are the influencing factors on tannins’ ability to precipitate protein [39].

## 4. Conclusions

The type of polymeric CT, the degree of polymerization, and the distribution of polymers were characterized by MALDI-TOF MS and NMR spectroscopy. The antioxidant activity assay showed that PCT possesses a strong antioxidant activity which is comparable with that of the synthetic antioxidant BHA. In addition, the binding interactions of the PCT with five metal ions (Cu^2+^, Zn^2+^, Al^3+^, Fe^2+^, and Fe^3+^) were characterized by a fluorescence quenching method. The results showed that a high molecular weight of polymeric CT up to tridecamers was detected by MALDI-TOF MS spectroscopy, and epicatechin was the basic unit occurring in the PCT. A-type and B-type linkages were the most common between the structural units of the CT polymers. The quenching experiment showed that Zn^2+^, Cu^2+^, and Al^3+^ had different mechanisms of quenching the PCT fluorescence intensity from Fe^3+^ and Fe^2+^. The affinities of Al^3+^ and Fe^3+^ for the PCT were much higher than those of Zn^2+^, Cu^2+^, and Fe^2+^. The affinities of the tested metals for the PCT could be ranked in the order Al^3+^ > Fe^3+^ > Zn^2+^ > Fe^2+^ > Cu^2+^, suggesting that trivalent metal ions exhibit higher affinities for condensed tannins than divalent metal ions. For all tested metal ions, the pH values greatly affected their binding interactions with PCT. Therefore, the data of the current research may help in the design of strategies to prevent metal-induced toxicity in individuals with a high exposure to transition metal ions. A simple UV-Vis spectra method was developed to determine the protein-precipitating capacity of tannins isolated from plum fruits, Chinese gallnut, sorghum grain, and *P. strobilacea*. The results showed that tannins isolated form Chinese gallnut have higher protein-precipitating capacity than tannins isolated from sorghum grain, *P. strobilacea*, and plum fruits.

## Figures and Tables

**Table 1:** Observed and calculated masses *^a^* of heteropolyflavan-3-ols by MALDI-TOF/MS.

Polymer	No. of A-Type *^b^* Bonds	No. of B-Type Bonds	Calculated [M+Cs]^+^	Observed [M+Cs]^+^
Dimer	0	1	711	711
Trimer	1	1	997	997
	2	0	995	995
Tetramer	1	2	1285	1285
	2	1	1283	1283
Pentamer	1	3	1573	1573
	2	2	1571	1571
Hexamer	1	4	1861	1860
	2	3	1859	1858
Heptamer	1	5	2149	2148
	2	4	2147	2146
Octamer	1	6	2437	2436
	2	5	2435	2435
Nonamer	1	7	2725	2724
	2	6	2723	2722
Decamer	2	7	3011	3011
	3	6	3009	3009
Undecamer	2	8	3299	3299
	3	7	3297	3297
Dodecamer	2	9	3587	3586
Tridecamer	2	10	3875	3875

*^a^* Mass calculations were based on the equation 290+288a+133, where 290 is the molecular weight of the terminal unit, a is the degree of polymerization (DP) contributed by the catechin extending unit, and 133 is the atomic weight of cesium. *^b^* The formation of each A-type interflavan ether linkage leads to the loss of two hydrogen atoms (2 amu).

**Table 2:** Stern–Volmer constants (*K*_a_) describing PCT fluorescence quenching by metal ions at pH 6.0.

	Concentration (μM)	*K*_a_ (×10^4^ L/mol)	*R*
Zn^2+ *a*^	<66.23	1.34	0.992
Cu^2+ *a*^	<66.23	1.00	0.999
Al^3+ *a*^	<33.22	7.65	0.999
Fe^2+ *b*^	<39.84	1.15	0.985
Fe^3+ *c*^	<33.22	6.48	0.989

*^a^* Stern–Volmer constants obtained by Equation (3); *^b^* Stern–Volmer constants obtained by Equation (4); *^c^* Stern–Volmer constants obtained by Equation (5).

## Data Availability

Data are contained within the article.

## Supplementary Materials

The following supporting information can be downloaded at: https://www.mdpi.com/article/10.3390/antiox11040714/s1. Figure S1: ^13^C NMR spectrum of condensed tannins extracted from plum pericarp. Figure S2: The quenching effects of Zn^2+^, Cu^2+^, Al^3+^, and Fe^3+^ on the PCT fluorescence intensity at a pH of 6.0 achieved with an acetate buffer at 294.82 K; *λ*_ex_ = 280 nm. Figure S3: The quenching effects of Al^3+^ and Fe^3+^ on the PCT fluorescence intensity at a pH of 2.1 or 7.4 at 294.82 K; *λ*_ex_ = 280 nm. Figure S4: The quenching efficiencies of different concentrations of Al^3+^ and Fe^3+^ on the PCT. The quenching efficiency was obtained by plotting [(*F*_0_ − *F*) × 100/*F*_0_] versus the metal ion concentration. Figure S5: Modified Stern–Volmer plots for the (a) Cu^2+^ and (b) Fe^2+^ quenching of the PCT fluorescence at a pH of 6.0. (c) Stern–Volmer plot for PCT fluorescence quenching by Al^3+^ or Fe^3+^ at a pH of 2.1 or 7.4. (d) Comparison of Stern–Volmer constants (*K*_a_) of PCT fluorescence quenching by Al^3+^ or Fe^3+^ at a pH of 2.1, 6.0, or 7.4.
