# Supplementary material for: Isolation, Characterization, Antioxidant Activity, Metal-Chelating Activity, and Protein-Precipitating Capacity of Condensed Tannins from Plum (Prunus salicina) Fruit
Source: Antioxidants (Basel). 2022 Apr 5;11(4):714. doi: 10.3390/antiox11040714 (PMC9030958; doi:10.3390/antiox11040714)
Supplement: Supplementary file 1 [file antioxidants-11-00714-s001.zip › antioxidants-1655770-supplementary.pdf]

Supplemental materials:

Figure S1. ^{13}C NMR spectrum of condensed tannins extracted from plum pericarp.

Figure S2. The quenching effects of Zn^{2+}, Cu^{2+}, Al^{3+}, and Fe^{3+} on the PCT fluorescence intensity at a pH of 6.0, achieved with an acetate buffer, at 294.82 K; λ_{ex} = 280 nm.

Figure S3. The quenching effects of Al^{3+} and Fe^{3+} on the PCT fluorescence intensity at a pH of 2.1 or 7.4 at 294.82 K; λ_{ex} = 280 nm.

Figure S4. The quenching efficiencies of different concentrations of Al^{3+} and Fe^{3+} on PCT. The quenching efficiency was obtained by plotting $[(F_0 - F) \times 100/F_0]$ versus the metal ion concentration.

Figure S5. Modified Stern–Volmer plots for quenching of the PCT fluorescence by (a) Cu^{2+} and (b) Fe^{2+} at a pH of 6.0. (c) Stern–Volmer plot for PCT fluorescence quenching by Al^{3+} or Fe^{3+} at a pH of 2.1 or 7.4. (d) Comparison of Stern–Volmer constants (K_a) of the PCT fluorescence quenching by Al^{3+} or Fe^{3+} at a pH of 2.1, 6.0, or 7.4.

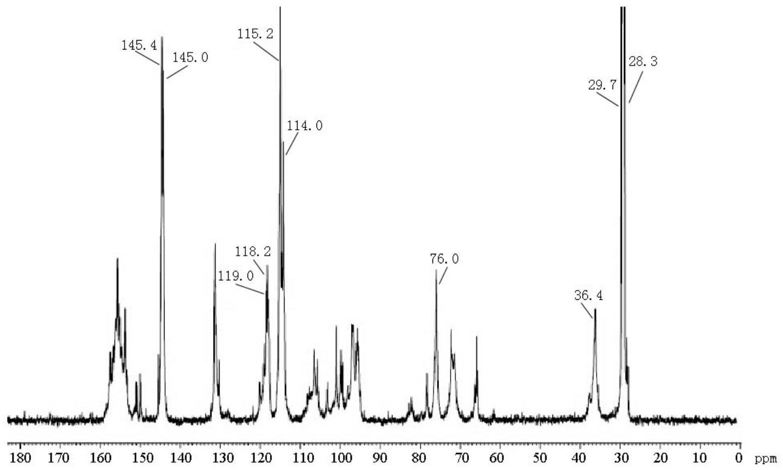
145.4
145.0
115.2
114.0
118.2
119.0
76.0
36.4
29.7
28.3
180 170 160 150 140 130 120 110 100 90 80 70 60 50 40 30 20 10 0 ppm

Figure S1

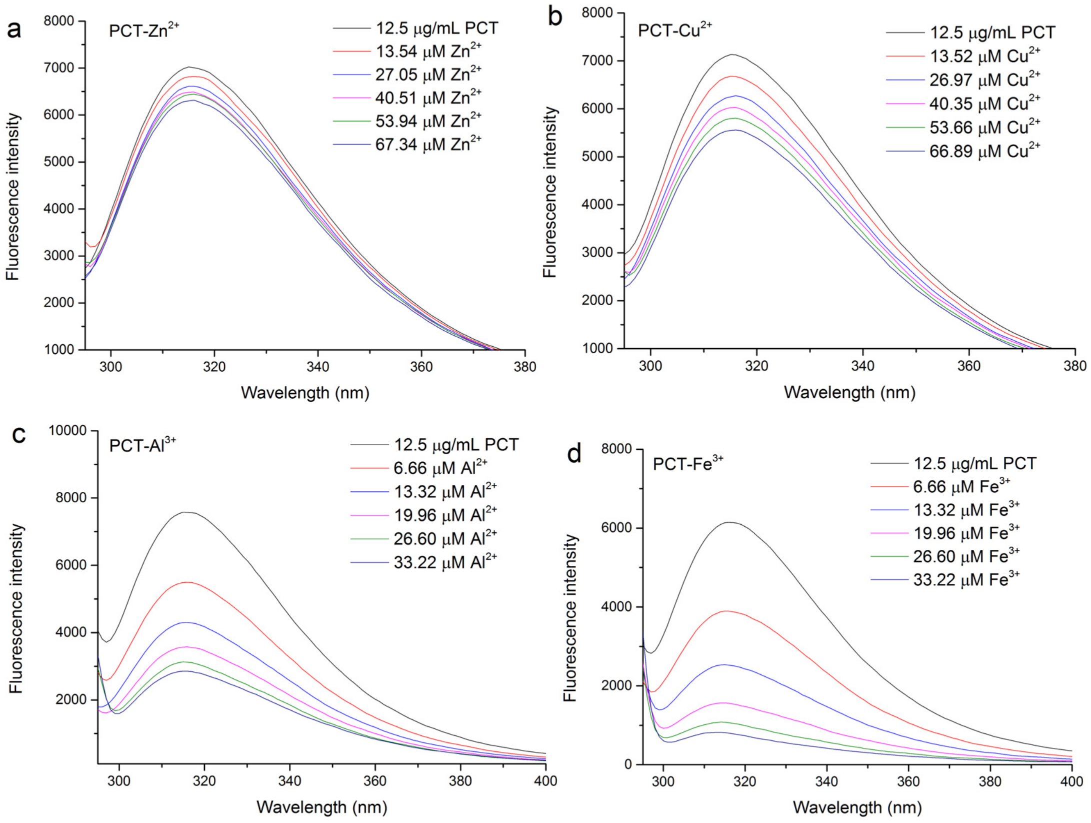
a
PCT-Zn2+
12.5 μg/mL PCT
13.54 μM Zn2+
27.05 μM Zn2+
40.51 μM Zn2+
53.94 μM Zn2+
67.34 μM Zn2+
Fluorescence intensity
Wavelength (nm)
b
PCT-Cu2+
12.5 μg/mL PCT
13.52 μM Cu2+
26.97 μM Cu2+
40.35 μM Cu2+
53.66 μM Cu2+
66.89 μM Cu2+
Fluorescence intensity
Wavelength (nm)
c
PCT-Al3+
12.5 μg/mL PCT
6.66 μM Al2+
13.32 μM Al2+
19.96 μM Al2+
26.60 μM Al2+
33.22 μM Al2+
Fluorescence intensity
Wavelength (nm)
d
PCT-Fe3+
12.5 μg/mL PCT
6.66 μM Fe3+
13.32 μM Fe3+
19.96 μM Fe3+
26.60 μM Fe3+
33.22 μM Fe3+
Fluorescence intensity
Wavelength (nm)

Figure S2

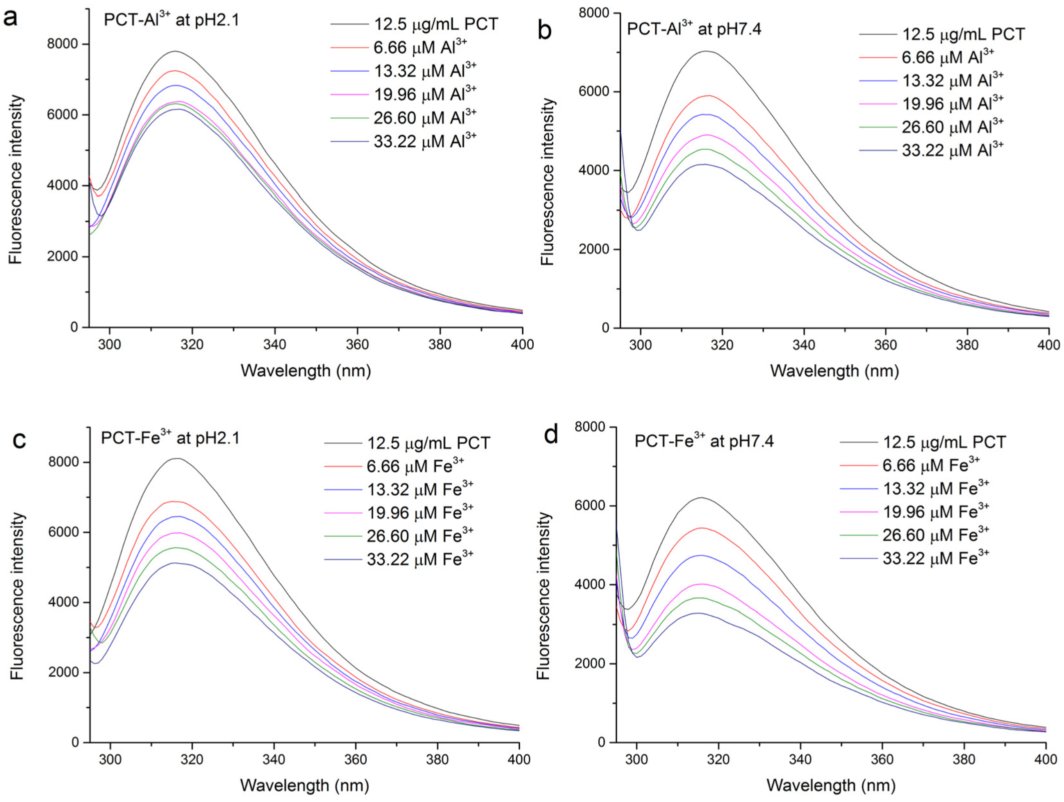

a
PCT-Al3+ at pH2.1
12.5 μg/mL PCT
6.66 μM Al3+
13.32 μM Al3+
19.96 μM Al3+
26.60 μM Al3+
33.22 μM Al3+
Fluorescence intensity
Wavelength (nm)
b
PCT-Al3+ at pH7.4
c
PCT-Fe3+ at pH2.1
12.5 μg/mL PCT
6.66 μM Fe3+
13.32 μM Fe3+
19.96 μM Fe3+
26.60 μM Fe3+
33.22 μM Fe3+
d
PCT-Fe3+ at pH7.4

Figure S3

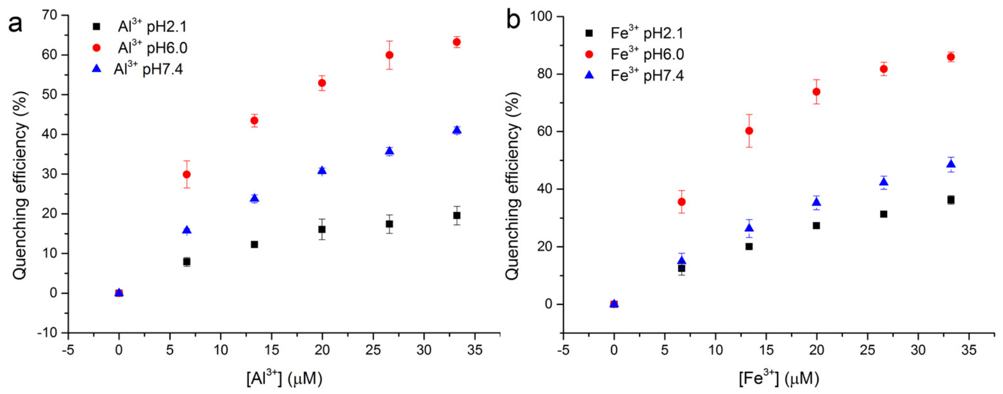

a
Al3+ pH2.1
Al3+ pH6.0
Al3+ pH7.4
Quenching efficiency (%)
[Al3+] (μM)
b
Fe3+ pH2.1
Fe3+ pH6.0
Fe3+ pH7.4
Quenching efficiency (%)
[Fe3+] (μM)

Figure S4

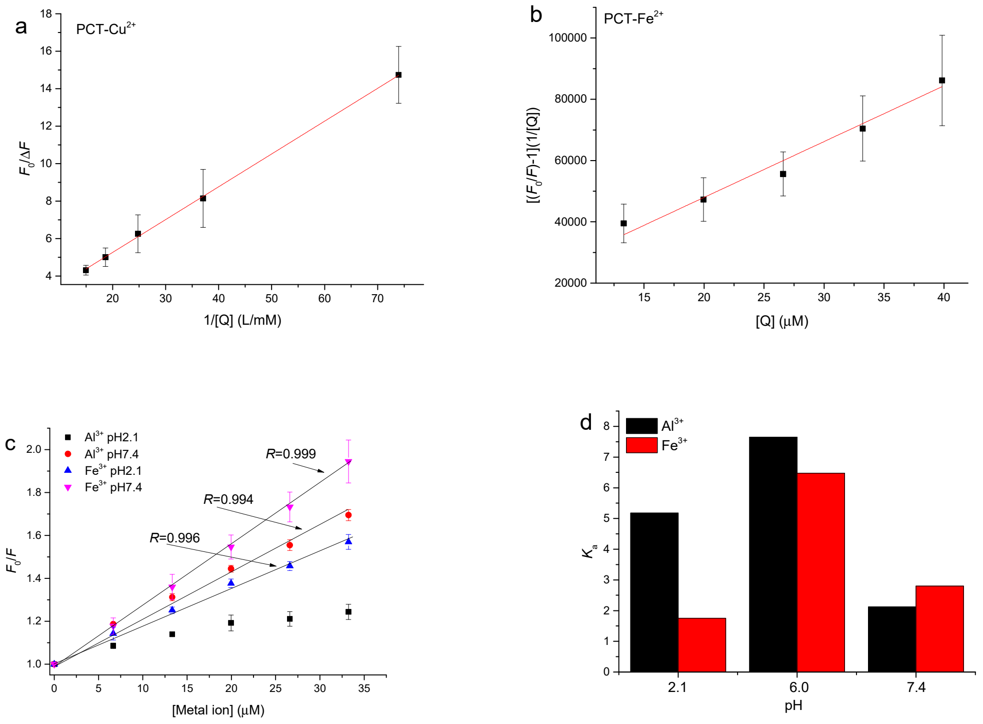
a
PCT-Cu2+
F0/ΔF
1/[Q] (L/mM)
b
PCT-Fe2+
[(F0/F)-1](1/[Q])
[Q] (μM)
c
Al3+ pH2.1
Al3+ pH7.4
Fe3+ pH2.1
Fe3+ pH7.4
R=0.999
R=0.994
R=0.996
F0/F
[Metal ion] (μM)
d
Al3+
Fe3+
Ka
pH

Figure S5
